# Focal Distal Esophageal Dilation (Blown-Out Myotomy) After Achalasia Treatment: Prevalence and Associated Symptoms

**DOI:** 10.14309/ajg.0000000000002816

**Published:** 2024-04-15

**Authors:** Thijs Kuipers, Fraukje A. Ponds, Paul Fockens, Barbara A.J. Bastiaansen, John E. Pandolfino, Albert J. Bredenoord

**Affiliations:** 1 Department of Gastroenterology and Hepatology, Amsterdam UMC Location University of Amsterdam, Amsterdam, the Netherlands;; 2 Amsterdam Gastroenterology, Endocrinology & Metabolism, Amsterdam, the Netherlands;; 3 Feinberg School of Medicine, Northwestern University, Chicago, Illinois, USA.

**Keywords:** achalasia, POEM, blownout myotomy

## Abstract

**INTRODUCTION:**

Peroral endoscopic myotomy (POEM) may result in a distended distal esophagus, referred to as a blown-out myotomy (BOM), the relevance of which is uncertain. The aim of this study was to investigate the prevalence, risk factors, and associated symptoms of BOM after achalasia treatment.

**METHODS:**

A data set of the locally treated patients in a randomized controlled trial comparing POEM with pneumatic dilation (PD) was analyzed. A BOM is defined as a >50% increase in esophageal diameter at its widest point in the distal esophagus between the lower esophageal sphincter and 5 cm above.

**RESULTS:**

Seventy-four patients were treated in our center, and 5-year follow-up data were available in 55 patients (32 patients [58%] randomized to POEM, 23 [42%] PD). In the group initially treated with POEM, the incidence of BOM increased from 11.5% (4/38) at 3 months, to 21.1% (8/38) at 1 year, 27.8% (10/36) at 2 years, and 31.3% (10/32) at 5 years. None of the patients treated with PD alone developed a BOM. Patients who developed a BOM had a higher total Eckardt score and Eckardt regurgitation component compared with patients who underwent POEM without BOM development (3 [2.75–3.25] vs 2 [1.75–3], *P* = 0.032, and 1 [0.75–1] vs 0 [0–1], *P* = 0.041). POEM patients with a BOM more often report reflux symptoms (85% [11/13] vs 46% [2/16], *P* = 0.023) and had a higher acid exposure time (24.5% [8–47] vs 6% [1.2–18.7], *P* = 0.027).

**DISCUSSION:**

Thirty percent of the patients treated with POEM develop a BOM, which is associated with a higher acid exposure, more reflux symptoms, and symptoms of regurgitation.

## INTRODUCTION

Achalasia is an esophageal motor disorder characterized by dysphagia, regurgitation, chest pain, and weight loss. These symptoms are the result of a combination of absent peristalsis and impaired relaxation of the lower esophageal sphincter (LES) (1). Currently there is no cure for achalasia. Treatment options focus on lowering the LES resting pressure with the aim to improve emptying of the esophagus and relief the symptoms. Available treatment options include pneumatic dilations (PDs), laparoscopic Heller myotomy (LHM), and peroral endoscopic myotomy (POEM) (2).

All treatment options are effective, and we found a success rate of a single series of dilations (30–35 mm) of 40%, allowing multiple redilations to improve the success rate. However, the variety in PD treatment protocols makes it difficult to compare the results. The success rate of LHM and POEM is reported to be 80%–90% (3,4). Treatment failure after LHM or POEM can have several causes; the main hypothesis is that failure is the result of incomplete myotomy or incomplete disruption of the LES (5). Another possible mechanism of treatment failure after myotomy is currently a point of interest as well. When focal dilation of a distal segment of the esophagus occurs after endoscopic or laparoscopic myotomy is performed, this is sometimes referred to as a blown-out myotomy (BOM) or pseudo-diverticulum. A pseudo-diverticulum or distal focal dilation at the myotomy site has been observed in a subset of patients with recurrent symptoms after myotomy (6–8). This phenomenon has also been described earlier after LHM (9–11). While some studies suggest a pseudo-diverticulum or distal focal dilation after myotomy is a normal postoperative radiographic finding, other studies found it is accompanied by more frequent and severe symptoms. In patients treated with a myotomy and partial fundoplication, a pseudo-diverticulum was present in approximately two-third of the patients and the presence was accompanied by increased symptoms of esophageal retention (11). More recently, a large cohort showed higher treatment failure rates in patients with BOM (based on Eckardt score >3) compared with patients without a BOM: 56.5% vs 29.2% (*P* = 0.009), respectively, after treatment with POEM or LHM (7). It should be noted there is a referral bias in this cited study performed at a tertiary referral center that might explain the high number of treatment failures in both groups.

The aim of this study was to evaluate the development of BOM over time and the occurrence of associated symptoms in the patient cohort that participated in our randomized controlled POEMA trial. This is a unique data set containing systematic evaluations at given time points in patients with treatment-naïve achalasia randomly assigned to either POEM or PD.

## METHODS

### Subjects

A data set of locally treated and followed patients in a randomized controlled trial comparing POEM with PD was analyzed (12). Owing to availability of the source data (original barium esophagram images), we only analyzed patients included in our center. In this original trial, newly diagnosed patients with symptomatic achalasia (based on an Eckardt greater than 3) were randomly allocated to undergo treatment with POEM or PD. Randomization was stratified per center, and no significant difference in treatment success rate on comparing the different centers was seen at the 5-year follow-up (4). Visits were scheduled at 3 months and at 1, 2, and 5 years after initial treatment. The following measurements were performed at all visits: Eckardt score and questionnaires (achalasia-specific quality-of-life questionnaire, Medical Outcomes Study 36-item Short Form Health Survey, and Gastroesophageal Reflux Disease Questionnaire), high-resolution manometry parameters, timed barium esophagram, and upper endoscopy. At the 1-year follow-up, 24-hour pH-impedance monitoring was performed in addition to the other measurements. In addition to these measurements, demographic data (age, sex, body mass index [BMI]), achalasia subtype, and procedural information was recorded.

### Treatment

Patients with treatment-naive achalasia were randomized to receive a single series of PD (30–35 mm) or POEM.

### Pneumatic dilation

A Rigiflex balloon (Boston Scientific) was positioned at the esophagogastric junction and dilated at a pressure of 5 psi for 1 minute, followed by dilation with 8 psi for another minute. Initial PD was performed using a 30 mm balloon. Evaluation of symptoms took place 3 weeks after initial treatment. If the Eckardt score exceeded 3, a second PD with a 35 mm balloon was performed. Conversely, if the Eckardt score was 3 or less, high-resolution manometry was conducted. If the integrated relaxation pressure (IRP) measured 10 mm Hg or higher, patients underwent a second PD with a 35 mm balloon.

### Peroral endoscopic myotomy

POEM was performed under general anesthesia with endotracheal intubation while patients were in a supine position. The procedure followed the protocol outlined by Inoue and colleagues (13). An endoscopic knife was used to access the submucosa, create the submucosal tunnel, and divide the circular muscle layer in the distal esophagus over a minimum length of 6 cm, extending 2–3 cm onto the cardia, including cutting the lower esophageal sphincter as per surgical myotomy standards. Standard endoscopic clips were used for closure of the mucosal entry site. Patients were admitted to the hospital either the day before or on the day of the procedure and discharged the following day if fluoroscopy revealed no evidence of leakage or perforation. The full details regarding the procedure can be found in the original article (12).

### BOM definition

We defined a BOM as a >50% increase in esophageal diameter at its widest point in the distal esophagus between the lower esophageal sphincter and 5 cm above as previously described (7). All available timed barium esophagrams were reviewed, and esophageal diameters were measured and recorded according to standard criteria by T.K. and A.B. (14). Barium column height at 1 and 5 minutes was also measured and registered. We also calculated the barium column surface (barium height × width) at both time points since this may be more representative for stasis in patients with a wider esophageal diameter. In patients with a wider esophageal diameter, the barium column height can be low while there is a significant amount of stasis.

### Clinical assessment

The Eckardt score was used to assesses the severity of achalasia symptoms. This score is the sum of symptom frequency scores for dysphagia, regurgitation, and chest pain combined with weight loss. Each question is graded on a 0 to 3 score resulting in a range of 0 (the lowest severity of symptoms) to 12 (the highest severity of symptoms). Scores below 3 are usually considered patients in remission (15).

### 24-hour pH-impedance measurement

Total acid exposure time (% of time with a pH < 4) and acid exposure time in upright and supine positions was registered, and a total acid exposure time of >6% was considered pathological (16). Analysis of all acid exposure episodes during the 24-hour pH-impedance measurements was conducted using the 5 previously distinguished acidification patterns in patients with achalasia. The following patterns are described: (A) acid reflux with normal clearance: a rapid decline in pH to below 4, with a drop rate of at least 1 pH unit per second, with a duration ranging from 10 seconds to 5 minutes; (B) acid reflux with delayed clearance: a rapid decline in pH to below 4, with a drop rate of at least 1 pH unit per second, with a duration of more than 5 minutes; (C) acid fermentation: distinguished by a gradual pH reduction to below 4, with a drop rate slower than 1 pH unit per minute with a duration of more than 5 minutes; (D) stasis of recently consumed acidic food or beverages: observed as a pH decline to below 4 during a meal or drink, and continues for more than 5 minutes after the meal or drink period; and (E) unclassified: characterized by a pH drop to below 4 that does not fulfill the criteria for any of the acid patterns described above (17).

### Statistical analysis

SPSS statistics (version 26; SPSS, Chicago, IL) was used for statistical analysis. Descriptive statistics were presented as percentage for categorical data, and continuous variables were presented as mean with SD or median with interquartile range. Analysis of categorical data was performed using χ^2^ or Fisher exact tests. Continuous data were compared using the unpaired Student *t* test for normal distributed data and Mann-Whitney *U* test for non-normally distributed data. A *P* value of <0.05 was considered significant.

## RESULTS

### Study patients

In total, 74 newly diagnosed achalasia patients were included in the POEMA trial in our center between October 2012 and July 2015. In 55 (74%) of the original 74 patients treated in our center, a barium esophagram was available at the 5-year follow-up: 32 patients (58%) randomized to POEM and 23 (42%) randomized to PD. We were only able to evaluate the occurrence of a BOM and the correlation with symptoms if the original images of the barium esophagram were available. Reasons for missing barium esophagram images at the 5-year follow-up are listed in Supplementary Table 1 (see Supplementary Digital Content 1, http://links.lww.com/AJG/D255). Baseline criteria including age, BMI, sex, achalasia subtype, pretreatment IRP-4, basal LES pressure, and barium column height were similar between both treatment groups, as shown in Table 1. During the 5-year follow-up, a part of the patients received re-treatment. In the patients initially treated with POEM, 7 (22%) of 32 received re-treatment and all of these patients were re-treated with PD. Amon the patients initially treated with PD, 17 (74%) of 23 received re-treatment, 9 (39%) of 23 received additional PD only, 6 (26%) of 23 received additional PD followed by POEM, and 2 (9%) of 23 received POEM directly after failure. In the end, 15 patients (27%) were treated with PD only and 40 (73%) were treated with POEM or PD + POEM. A BOM was seen in none of the patients treated with PD alone, and 32.5% (13/40) of the patients ever treated with POEM at 5-year follow-up (*P* = 0.011). Since a BOM did not occur in any patients treated with PD only, we focused on patients initially or ever treated with POEM in the remaining results. Esophageal diameter on barium esophagram before treatment was wider in the group without BOM compared with the patients with a BOM in patients ever treated with POEM.

**Table 1.**
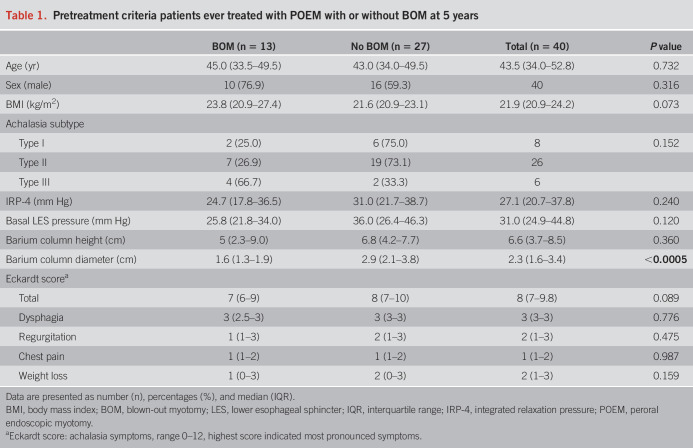
Pretreatment criteria patients ever treated with POEM with or without BOM at 5 years

### Follow-up

**Figure 1.**
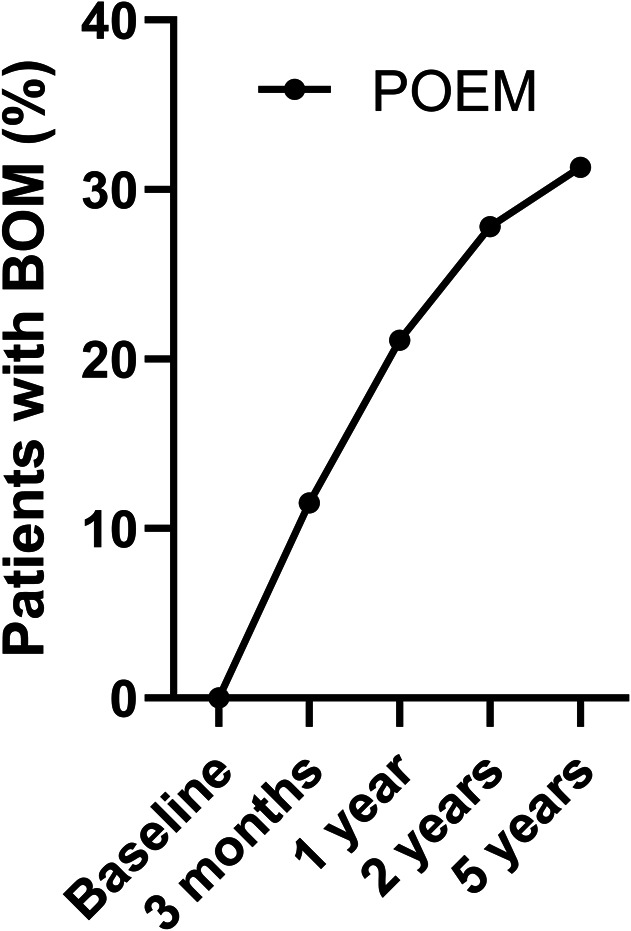
Percentage of patients initially treated with POEM with a BOM on barium esophagogram during the course of the follow-up. BOM, blown-out myotomy; POEM, peroral endoscopic myotomy.

**Figure 2.**
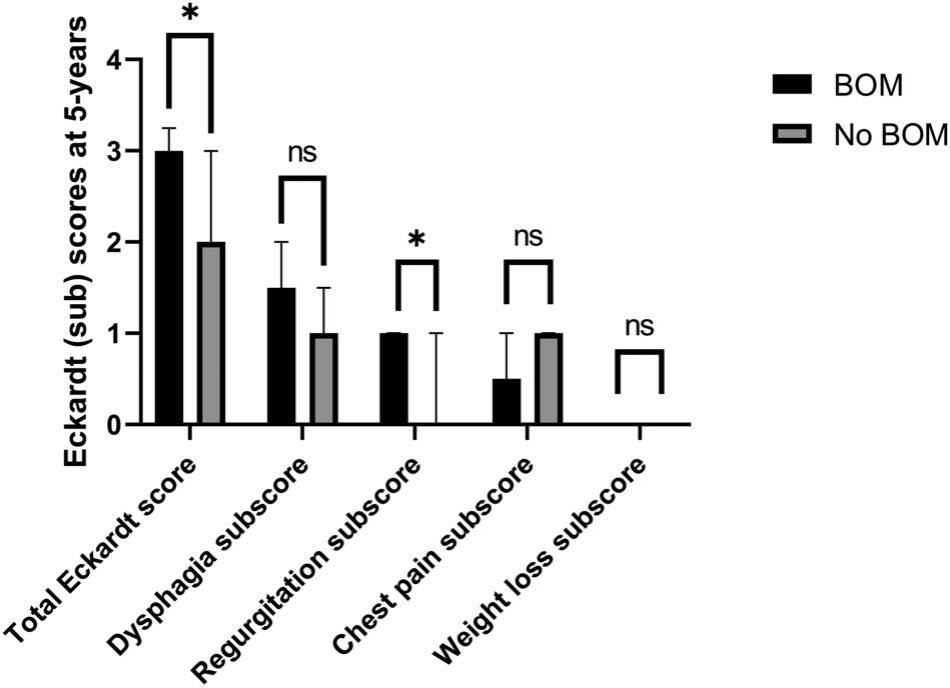
Total Eckardt score and Eckardt subscores at 5-year follow-up in patients initially treated with POEM with or without BOM. BOM, blown-out myotomy; POEM, peroral endoscopic myotomy.

Before treatment, no pseudo-diverticulum was visible on the barium esophagram in any of the patients that participated in the trial. During the course of the follow-up, the incidence of a BOM increased from 11.5% (4/38) at 3 months, to 21.1% (8/38) at 1 year, 27.8% (10/36) at 2 years, and 31.3% (10/32) at 5 years in patients initially treated with POEM, as shown in Figure 1. Barium column height at 1 minute (3.5 [2.0–5.8] cm vs 4 [1.5–5.4] cm, *P* = 0.857) and 5 minutes (3.2 [0–5.6] cm vs 3.3 [0.8–4.1], *P* = 0.952) was comparable in patients with or without a BOM. The barium column surface (barium height × width) was not significantly different at 1 minute (12.2 [8.4–19.1] cm^2^ vs 10.5 [3.0–17.6] cm^2^, *P* = 0.345) or at 5 minutes (10.8 [2.7–18.9] cm^2^ vs 7.2 [0–12.8] cm^2^, *P* = 0.197) in patients with or without a BOM. In this group, patients with a BOM had a higher total Eckardt score and Eckardt regurgitation subscore (3 [2.75–3.25] vs 2 [1.75–3], *P* = 0.032, 1 [0.75–1] vs 0 [0–1], *P* = 0.041, respectively) compared with patients without a BOM at the 5-year follow-up. The Eckardt dysphagia, chest pain, and weight loss subscores were not different in both groups (Figure 2). In addition, at 5-year follow-up, no significant difference was seen in IRP-4 (13.4 [9.1–18.8] mm Hg vs 12.1 [8.5–16.7] mm Hg, *P* = 0.377) and LES resting rate (18.1 [14.3–26.0] mm Hg vs 22.3 [13.7–29.8] mm Hg, *P* = 0.642) in patients with or without a BOM.

### Reflux

Patients more often reported reflux symptoms (based on Gastroesophageal Reflux Disease Questionnaire ≥8) when a BOM was present compared with when no BOM was present (85% [11/13] vs 46% [2/16], *P* = 0.023). A BOM did not have an effect on the presence of reflux esophagitis (54% [7/13] vs 41% [11/27], *P* = 0.329) or PPI use (54% [7/13] vs 44% [12/27], *P* = 0.413). Acid exposure time (measured at 1-year follow-up) was significantly higher in patients with a BOM compared with no BOM (24.5% [8–47] vs 6% [1.2–18.7], *P* = 0.027). Pathological acid exposure (acid exposure time >6%) was seen in 7 of 9 patients (77.8%) with a BOM and 20 of 38 (52.6%) (*P* = 0.170) without. Analysis of the acidification patterns showed that acid reflux with delayed clearance was seen more often in patients with a BOM compared with those without a BOM (2.7% [0.2–20.2] vs 0% [0–1.8], *P* = 0.023), which resulted in a higher total percentage of time with a pH below 4. Also the number of acidic events caused by acid reflux with delayed clearance was significantly higher (1.5 [0.3–3.8] vs 0 [0–1], *P* = 0.015) in the patients with a BOM. Full analysis of the different acidification patterns is presented in Table 2.

**Table 2.**
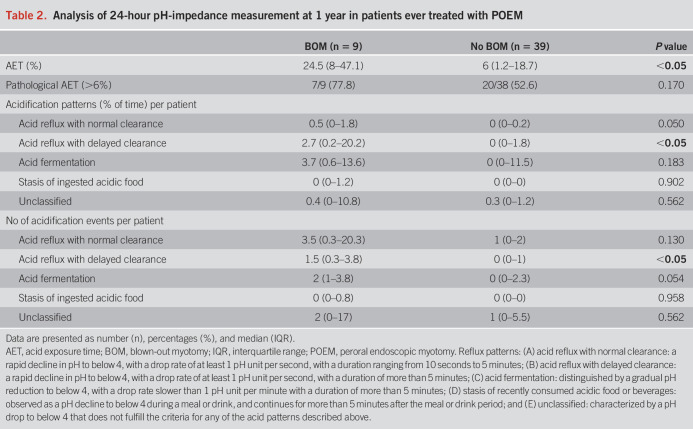
Analysis of 24-hour pH-impedance measurement at 1 year in patients ever treated with POEM

### Quality of life

Patients with a BOM scored 17.5 (14.0–20.0) on the achalasia-specific quality-of-life questionnaire, and patients without BOM scored 17.0 (13.0–18.5, *P* = 0.287). Both the 36-item Short Form Health Survey mental component (56.0 [53.6–59.6] vs 56.8 [50.7–59.8], *P* = 0.767) and physical component (52.9 [48.6–55.3] vs 52.7 [47.6–56.4], *P* = 1.000) scores did not significantly differ in patients with or without a BOM.

### Risk factors

Achalasia subtype did seem to be a risk factor for the occurrence of a BOM in our cohort, as BOM occurred in 25% (2/8) of type 1, 27% (7/26) of type II, and 67% (4/6) of type III achalasia (*P* = 0.152). Myotomy length (12.5 [11.8–16.5] cm vs 13.5 [12–17.3] cm, *P* = 0.735) did not significantly differ in patients with or without a BOM. Patients with subtype I achalasia had a median myotomy length of 13.5 (12.8–20) cm, subtype II a length of 13 (11.3–15) cm, and subtype III a length of 17.5 (12.5–20) cm (*P* = 0.160). Other baseline criteria such as sex, BMI, manometric features (LES resting pressure, IRP-4), and barium column height did not differ in patients with or without a BOM. In addition, we evaluated the first available manometries after treatment with POEM and analyzed the residual pressure in the part proximal to the myotomy. In patients who developed a BOM over time, the residual pressure was 122 (14–221) mm Hg·s·cm compared with 49 (1–88) mm Hg·s·cm in patients who did not develop a BOM (*P* = 0.200). Three months after treatment with POEM, the IRP-4 (14 [7.6–20.5] mm Hg vs 11.4 [5.4–16.1] mm Hg, *P* = 0.238) and LES resting pressure (13.8 [11–14.5] vs 9 [6.7–18.1], *P* = 0.696) were not significantly different in patients developing a BOM vs patients without a BOM.

We did not see a difference in BOM development as endosurgical experience with POEM improved. The first 10 vs later treated (*P* = 0.397) or first 20 vs later treated (*P* = 0.135) patients with POEM in this trial were not more likely to develop a BOM compared with the patients treated thereafter.

## DISCUSSION

We have evaluated the incidence and risk factors of development of a BOM after achalasia treatment, as well as corresponding symptoms using a part of the data set of our previously conducted randomized controlled trial. We found 30% of the patients treated with a POEM developed a BOM over time, while none of the patients treated with PD alone developed a BOM. A BOM was associated with a higher esophageal acid exposure, more reflux symptoms, and more frequent symptoms of regurgitation. It should be stressed, however, that most patients have a good response to POEM, the efficacy is considerably higher than PD, and the chance of developing a BOM is not a reason in itself to prefer PD over POEM. However, the presence of a BOM can be an explanation for certain symptoms patients develop in the years after a myotomy.

The observed prevalence of a BOM of 32.5% in the patients treated with POEM appears to be higher in our series compared with what has been reported previously. Only one other study reported incidence of a BOM after POEM, and this occurred in 9.4% of the patients but with a shorter follow-up of 14.9 months (7). The incidence of a BOM in patients treated with LHM is more frequently described and is seen in 15%–67% of the patients after LHM (10,11). If we compare our numbers with the incidence of a BOM after POEM, it is important to note that the median follow-up in our cohort was 60 months compared with 14.9 months in previously reported study (7). Since we do see an increase in incidence over the duration of follow-up, this might be the explanation for the difference in occurrence of a BOM after POEM. The studies that reported a high incidence of BOM after LHM (67%) had a follow-up of 86 months, and this could explain the higher number of patients with a BOM in that report (11). We found the development of a BOM was accompanied with more frequent esophageal symptoms in general and more symptoms of regurgitation in particular. This finding is in accordance with what was seen earlier after LHM (10). Other studies described significantly more symptoms of dysphagia and chest pain in addition to more symptoms of regurgitation based on the Eckardt subscore, which we did not find in our series (7).

The development of a BOM is associated with increasing esophageal acid exposure (24.5% vs 6%). It has been described that only reviewing acid exposure gives an overestimation of reflux in patients treated for achalasia (17). Differentiating between true acid reflux and other acidification patterns such as delayed clearance or fermentation is necessary (18). Detailed analysis of the pH measurements revealed the difference in acid exposure is mainly caused by delayed clearance. It suggests that the higher number reflux symptoms in these patients are the result of delayed reflux clearance in the pseudo-diverticulum. The combination of pseudo-diverticulum, increased regurgitation symptoms, and higher number of reflux symptoms is in line with what has been observed in patients after LHM (10). It should be noted that the incidence of a BOM seems higher in achalasia subtype III (67% compared with 25% and 27% in achalasia subtypes I and II, respectively); however, it did not reach statistical significance. A previous study did find achalasia type III to be significantly more associated with the development of a BOM (7).

One of the strengths of this study is the fact that we studied the presence of a BOM using a well-defined randomized controlled trial data set, in which well-characterized patients were included and measured at given time points. Follow-up was performed in all patients according to the study protocol and regardless of symptoms limiting risk for selection bias. Unfortunately and unpreventably, in a part of the patients, no barium esophagram was available at the 5-year follow-up. However, since reasons are diverse, selection bias seems limited. Another strength of this study is the detailed analysis of the 24-hour pH-impedance study in all patients, including those without complaints. This gives more insight into the nature of increased acid exposure in the presence of a BOM and whether this is associated with symptoms.

It is hypothesized that the development of a BOM is the result of a weakness in the esophageal wall at the point of the myotomy and persistent residual contractions as appropriate to achalasia. Over time, the stress will lead to deformation of the esophageal wall and lead to widening of the distal esophagus and formation of a pseudo-diverticulum. This hypothesis is supported by the fact that an epiphrenic diverticulum is very often secondary to an esophageal motility disorder and associated with a congenital weakness of the esophageal wall (19). The pseudo-diverticulum will have impaired emptying, cause delayed clearance, and induce regurgitation. Based on this mechanism, a reduction of outflow stress by lowering esophageal pressure or making the weak spot (myotomy site) less weak may be able to prevent/reduce the development of a BOM and reduce symptoms. Perhaps, reducing the vulnerability of the esophageal wall can be achieved by reducing the length of the myotomy or performing a selective circular myotomy instead of a full-thickness myotomy (20). A figure explaining this hypothesis is shown in Supplementary Figure 1 (see Supplementary Digital Content 1, http://links.lww.com/AJG/D255). We did find that a more narrow esophageal caliber before treatment is associated with a higher rate of BOM development. It can be hypothesized that patients with a wider caliber of the esophagus are in a more chronic state of achalasia where the esophageal body is already dilated/decompensated and the esophageal body muscle is weak. Patients with a narrow caliber of the esophagus may have more powerful residual contractions. Over time, it is more likely in these patients to develop a BOM at the myotomy side due to the powerful residual contractions (more stress over time on the esophageal wall).

Currently, we do not know if re-treatment is beneficial in patients with a BOM, and if so, what type of treatment is best used in patients with recurrent symptoms and a BOM on barium esophagram. More research has to be performed to give a good recommendation regarding re-treatment in case of recurrent symptoms and a BOM.

Based on this and previous studies, it is not known if development of a BOM is avoidable. It could be that a part of the patients treated with a POEM developed a BOM regardless of the type of myotomy used due to, as of now, unknown reasons, and the occurrence of a BOM should be seen more as explanation of recurrent symptoms. A randomized trial comparing a selective circular myotomy and a full-thickness myotomy could give more insight into this.

This study showed that with time, 30% of patients develop a BOM after POEM and a BOM is not seen after PD. The incidence of a BOM increases over time and is accompanied by more symptoms of regurgitation. These findings suggest that a BOM may be an important explanation for recurrent symptoms.

## CONFLICTS OF INTEREST

**Guarantor of the article:** Thijs Kuipers, MD.

**Specific author contributions:** T.K., J.P., and A.B. played a role in planning of the study. T.K. and A.B. had a role in conducting the study. T.K. was involved in the acquisition of data. T.K. and A.B. had a role in collecting and/or interpreting data. T.K., J.P., and A.B. played a role in drafting the manuscript. F.P., P.F., and B.B. played a role in reviewing and revising the manuscript for important intellectual content. All authors approved the final draft submitted.

**Financial support:** None to report.

**Potential competing interests:** T.K., F.P., and B.B. have no financial or personal competing interests. P.F. received consultancy fees from Olympus and Cook Medical. J.E.P.: Sandhill Scientific/Diversatek (Consulting, Speaking, Grant), Takeda (Speaking), Astra Zeneca (Speaking), Medtronic (Speaking, Consulting, Patent, License), Torax (Speaking, Consulting), Ironwood (Consulting). A.B. received research funding from Nutricia, SST, Thelial, Sanofi, Dr. Falk Pharma, and received speaker and/or consulting fees from Laborie, Medtronic, Dr. Falk Pharma, Calypso Biotech, Alimentiv, Regeneron/Sanofi, AstraZeneca.

**Study Highlights**

**WHAT IS KNOWN**

✓ POEM has proven to be an effective treatment option in achalasia.
✓ A blown-out myotomy (BOM) or pseudo-diverticulum has been observed after POEM.

**WHAT IS NEW HERE**

✓ Thirty percent of the patients treated with POEM develop a BOM.
✓ A BOM is accompanied with a higher acid exposure, more reflux symptoms, and symptoms of regurgitation.
✓ A BOM may be an important explanation for recurrent symptoms.

## Supplementary Material
