# Supplementary material for: Focal Distal Esophageal Dilation (Blown-Out Myotomy) After Achalasia Treatment: Prevalence and Associated Symptoms
Source: Am J Gastroenterol. 2024 Apr 15;119(10):1983–9. doi: 10.14309/ajg.0000000000002816 (PMC11446521; doi:10.14309/ajg.0000000000002816)

Supplemental material

| Esophagram performed, not available in electronic health record | N=1 |
| --- | --- |
| Patient does not want to visit | N=4 |
| Esophagram performed at 4 years follow-up | N=2 |
| Lost to follow-up due to stroke | N=2 |
| Lost to follow-up due to dementia | N=1 |
| Lost to follow-up reason unknown | N=1 |
| Patient died | N=1 |
| Patient not able to visit the hospital due concomitant disease | N=2 |
| Esophagram not performed, technical failure at radiology department | N=1 |
| Esophagram not performed due to COVID pandemic | N=1 |
| Patient moved to a foreign country | N=1 |
| Patient in follow-up, reason esophagram missing is unknown | N=2 |

**Supplementary table 1**; reasons unavailability barium esophagram


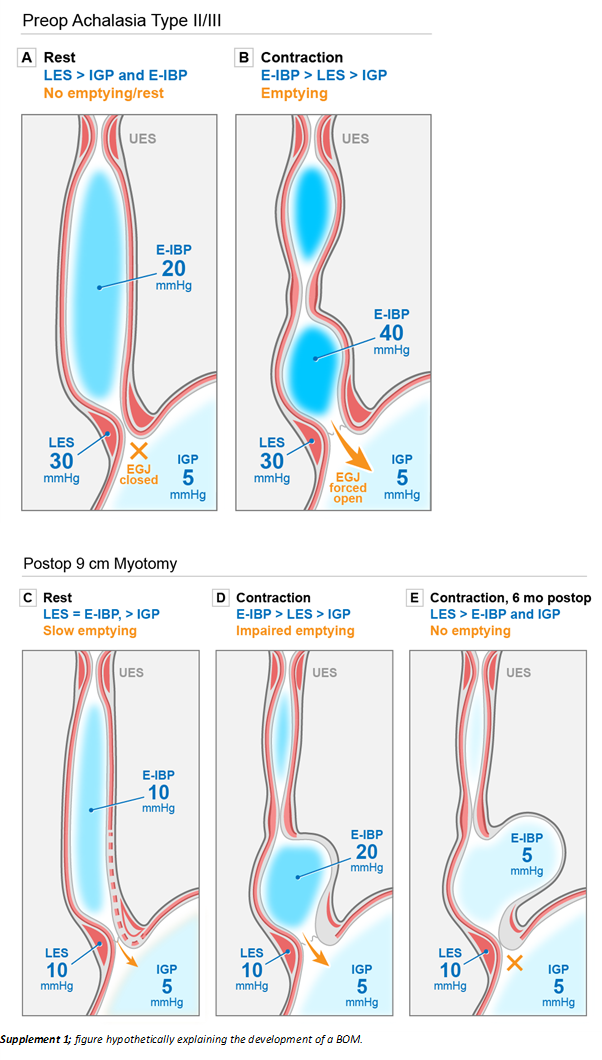

Supplement: Supplementary file 1 [file acg-119-1983-s001.docx]
